# Fatigue in patients with neuromyelitis optica spectrum disorder and its impact on quality of life

**DOI:** 10.1371/journal.pone.0177230

**Published:** 2017-05-23

**Authors:** Jin Myoung Seok, Misong Choi, Eun Bin Cho, Hye Lim Lee, Byoung Joon Kim, Kwang Ho Lee, Pamela Song, Eun Yeon Joo, Ju-Hong Min

**Affiliations:** 1 Department of Neurology, Samsung Medical Center, Sungkyunkwan University School of Medicine, Seoul, Korea; 2 Neuroscience Center, Samsung Medical Center, Seoul, Korea; 3 Department of Neurology, Soonchunhyang University College of Medicine, Cheonan Hospital, Cheonan, Korea; 4 Department of Neurology, Gyeongsang National University Changwon Hospital, Gyeongsang National University School of Medicine, Changwon, Korea; 5 Department of Neurology, Korea University Guro Hospital and Korea University College of Medicine, Seoul, Korea; 6 Department of Neurology, Ilsan Paik Hospital, Inje University College of Medicine, Goyang, Korea; Charite Universitatsmedizin Berlin, GERMANY

## Abstract

Fatigue is a prevalent symptom and major burden in neuroimmunological diseases. In neuromyelitis optica spectrum disorder (NMOSD), a severe autoimmune central nervous system (CNS) inflammatory disease with autoantibodies reactive to aquaporin-4, there are few reports about fatigue and quality of life (QOL). We aimed to evaluate the severity of fatigue and its relationship with QOL in patients with NMOSD. We prospectively studied patients with NMOSD who were in remission and seropositive for anti-aquaporin-4 antibody, and they were divided into 2 groups based on the presence of fatigue assessed using the Functional Assessment of Chronic Illness Therapy-fatigue score. Sleep quality, depression, pain, and QOL were also evaluated. A total of 35 patients were enrolled (mean age, 46.5 ± 14.1 years; female: male = 29:6), and the median Expanded Disability Status Scale (EDSS) score was 2.0 (range, 0 to 8.0). The patients with fatigue (N = 25, 71.4%) had poorer sleep quality and more severe depression than those without fatigue (p = 0.009 and p = 0.001). Both the physical and mental QOL scores were lower in patients with fatigue than in those without fatigue (p = 0.033 and p = 0.004). Multiple linear regression analyses showed that the degree of fatigue with EDSS score and pain were independent predictors of physical aspects of QOL (B = 0.382, p = 0.001), whereas depression was the only predictor of the mental components of QOL (B = -0.845, p = <0.001). Fatigue is a common symptom and an important predictor of QOL in patients with NMOSD.

## Introduction

Neuromyelitis optica (NMO) is an autoimmune relapsing inflammatory disorder of the central nervous system (CNS) characterized by optic neuritis, myelitis, and distinctive brain lesions.[1] An antibody against the main water channel protein in CNS, aquaporin-4 (anti-AQP4), is thought to be pathogenic and detected in 60–80% of patients with NMO.[2] NMO is distinguished from multiple sclerosis (MS) by its pathogenesis and clinical features;[1, 2] generally, it is regarded that patients with NMO have more severe attacks than relapsing-remitting MS.[1] There has been many studies on the impairment of quality of life (QOL) in MS in which fatigue, depression, and sleep disorder were considerable predictors of low QOL scores.[3–5] Particularly, fatigue was reported as one of the most frequent and disabling symptom in MS, and the improvement of fatigue is being considered as an important treatment target in patients with MS.[6, 7] In NMO, a recent study showed that the disease had a strong negative impact on health-related QOL in patients;[8] however, there are only a few reports on the relationship of fatigue and QOL in NMO. To date, the immunosuppressive therapy to reduce the number of relapses was the only apparent treatment goal of NMO,[9] although the impairment of QOL, severe fatigue, depression, and pain can be a major part of the burden of this disorder. In this study, we aimed to evaluate the severity of fatigue and its relationship with QOL in patients with NMO spectrum disorder (NMOSD).

## Materials and methods

### Patients

We prospectively studied consecutive NMO or NMOSD patients who were registered in the CNS Inflammatory Disease Registry at the Samsung Medical Center from June 2014 to May 2015. Patients were enrolled if they met the revised criteria for NMO or the suggestion of NMOSD with positive anti-AQP4,[1, 10] which is in agreement with the diagnosis of NMOSD with anti-AQP4 based on the International Consensus Diagnostic Criteria,[11] and they were during remission for at least 6 months. Patients who were excluded from the study are as follows: (a) anti-AQP4 status was either negative or not assessed using the cell-based indirect immunofluorescence assay as described previously,[12] (b) patients who refused to participate in the study, (c) patients who had medical disorders that could alter the status of fatigue including thyroid disease and adrenal insufficiency. The demographic and clinical characteristics were collected, including age, gender, disease duration, disability assessed by Expanded Disability Status Scale (EDSS) scores, current treatments such as immunosuppressants or oral prednisolone, and coexisting autoimmune diseases. All enrolled patients completed the self-report questionnaires for the assessment of fatigue, sleep quality, depression, pain, and QOL. The study was approved by the local ethics committees in Samsung Medical Center, and all participants provided written informed consent prior to the study.

### Instruments

The severity of fatigue was assessed with a Korean version of the Functional Assessment of Chronic Illness Therapy-fatigue scale (FACIT-fatigue), which is a 13-item questionnaire that assesses self-reported fatigue and difficulty of daily activities due to fatigue; its final scores range from 0 to 52, and higher scores indicate less fatigue.[13] This questionnaire was originally developed for the assessment of fatigue in patients with cancer; however, it has been validated and used in patients with autoimmune disease including Crohn’s disease, rheumatoid arthritis, systemic lupus erythematosus, and Sjogren’s syndrome.[14, 15] Considering relationship between NMOSD and systemic rheumatologic autoimmune diseases, using the FACIT-fatigue scale could be reasonable because the fatigue questionnaire specifically for the patients with NMOSD has not been settled yet.

The median score of FACIT-fatigue from a general population study in the United States with 1075 subjects was 43.[16] The patients were divided into 2 groups based on the FACIT-fatigue score: NMOSD without fatigue group (FACIT-fatigue score above 43) and NMOSD with fatigue group (FACIT-fatigue score of 43 or less).

Depression was evaluated by the Beck Depression Inventory score-II (BDI), which is composed of 21 items, and ranges from 0 to 63; higher scores indicate more severe depression. The Brief Pain Inventory (BPI), used for the assessment of pain has questions regarding 2 categories: pain severity and pain-related interference in daily life (each category ranges from 0 to 10); pain severity index score was evaluated by the average score of pain severity questions. The Pittsburgh Sleep Quality Index score (PSQI), which is composed of 7 items, and ranges from 0 to 21, was used for measuring the sleep quality. Higher scores of PSQI indicate poor sleep quality, and a poor sleeper was defined as a patient with PSQI score above 5. QOL was assessed via the Short Form 36 Health Survey (SF-36) with scores from 0 to 100, where higher scores indicate better QOL. Two summary scores of SF-36 were used for analysis: Physical component summary (PCS) and Mental component summary (MCS).

### Statistical analysis

Appropriate summary statistics were used to describe categorical and continuous variables; continuous data are shown as mean with standard deviation (SD) or median with inter-quartile range (IQR); categorical variables are presented with absolute and relative frequencies. We analyzed the differences between the groups (without fatigue versus with fatigue) using the Chi-square test or Fisher’s exact test for categorical variables; Student t-test or Mann-Whitney U test for cotinuous variables. Correlations between scores of the questionnaires were assessed using Spearman’s rho, and multiple linear regression analysis was performed to evaluate the independent contribution of factors that influenced the QOL; age, disease duration, EDSS, FACIT-fatigue, BDI, PSQI score, and pain severity index were included as possible independent variables for each model. Regression coefficients with 95% confidence intervals and their significance from multiple linear regression analysis were given. The multicollinearity was not observed among variables. Differences were considered significant at a value of *p* < 0.05. All statistical analyses were performed using commercially available software (SPSS for Windows, version 19.0; SPSS Inc., Chicago, IL, USA).

## Results

### Patient characteristics

A total of 35 patients who were seropositive for anti-AQP4 (mean age, 46.5 ± 14.1 years; female:male = 29:6) were finally enrolled (Table 1). 5 patients were excluded; two patients with recurrent optic neuritis and two patients with LETM were seronegative for anti-AQP4 antibody, and one patient was finally diagnosed as multiple sclerosis. The mean age of excluded patients was 44.6 ± 11.5 years which was not significantly different from those of enrolled patients (*P* = 0.771). The median disease duration was 3.0 years (IQR, 1.0 to 5.5 years), median EDSS score was 2.0 (IQR, 1.0 to 3.5), and the mean annualized relapse rate (ARR) was 1.6 ± 1.6. The most common clinical manifestation during the disease course was myelitis (N = 26, 74.3%), followed by optic neuritis (N = 20, 57.1%), brainstem or cerebellar syndrome (N = 10, 28.6%), and cerebral or diencephalic syndrome (N = 7, 20%). Demographic and clinical features such as age, sex ratio, disease duration, ARR, EDSS score, lesions of involvement, and preventive treatment including prednisolone and immunosuppressants were not different between patients with NMSOD with and without fatigue (all, *p* > 0.05). Thee coexistence of other autoimmune diseases was observed in 7 patients (20.0%) (N = 3, Sjogren’s syndrome; N = 3, systemic lupus erythematosus; and N = 1, primary antiphospholipid antibody syndrome).

**Table 1:** Clinical characteristics of patients with NMOSD based on the presence of fatigue.

	Total (N = 35)	NMOSD without fatigue (N = 10)	NMOSD with fatigue (N = 25)	*p*
Age, year (SD)	46.5 (14.1)	48.0 (18.2)	46.0 (12.5)	0.751
Female, n (%)	29 (82.9)	8 (80.0)	21 (84.0)	0.777
Median disease duration, year (IQR)	3.0 (1.0–5.5)	2.4 (0.4–6.0)	3.0 (1.1–5.3)	0.397
Number of relapses, (IQR)	2.0 (1.0–4.0)	2.0 (1.0–3.0)	2.0 (1.0–4.0)	0.986
EDSS score, (IQR)	2.0 (1.0–3.5)	2.0 (1.0–3.0)	3.0 (1.5–4.0)	0.097
Coexisting autoimmune diseases, n (%)	7 (20.0)	1 (10.0)	6 (24.0)	0.644
CNS involvement, n (%)				
Optic nerve	20 (57.1)	6 (60.0)	14 (56.0)	0.829
Spinal cord	26 (74.3)	5 (50.0)	21 (84.0)	0.081
Brain	14 (40.0)	2 (20.0)	12 (48.0)	0.252
Brainstem and cerebellum	10 (28.6)	2 (20.0)	8 (32.0)	0.686
Cerebrum and diencephalon	7 (20.0)	0	7 (28.0)	0.084
Coexisting spinal cord and brain	10 (28.6)	0 (0)	10 (40.0)	0.034
Preventive treatment, n (%)				0.880
Azathioprine	15 (42.9)	4 (40.0)	11 (44.0)	
Mycophenolate mofetil	8 (22.9)	2 (20.0)	6 (24.0)	
Oral prednisolone	3 (8.6)	1 (10.0)	2 (8.0)	
Other	5 (14.3)	1 (10.0)	4 (16.0)	

NMOSD, neuromyelitis optica spectrum disorder; SD, standard deviation; IQR, inter-quartile range; EDSS, Expanded Disability Status Scale; CNS, central nervous system.

### Self-reported questionnaires for fatigue, sleep quality, depression, and pain

**Fig 1:**
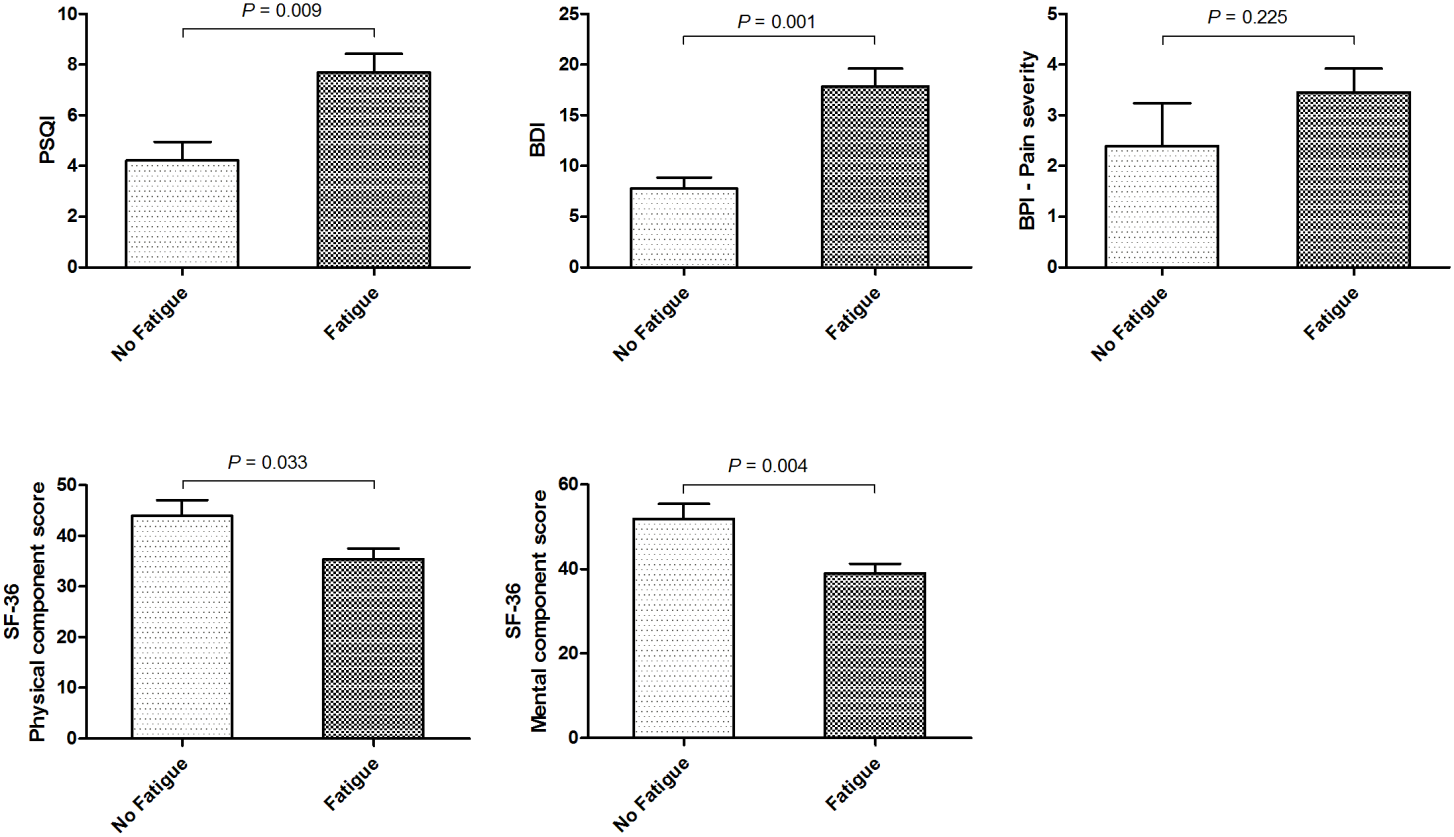
The differences in sleep quality, depression, pain severity, and quality of life scores in patients with NMOSD based on the presence of fatigue. PSQI, BDI, and BPI scores were higher in patients with NMSOD and fatigue than in those without fatigue. Moreover, the physical and mental component scores of quality of life assessed by SF-36 were lower in patients with NMSOD and fatigue. PSQI, Pittsburgh Sleep Quality Index; BDI, Beck Depression Inventory; BPI, Brief Pain Inventory; SF-36, Short-Form 36 Health Survey.

Results of self-administered questionnaires are summarized in Table 2. Mean FACIT-fatigue score was 32.9 ± 13.4 in a total of 35 patients; 71.4% (N = 25) had fatigue (mean FACIT-fatigue score, 27.6 ± 12.2), whereas 28.6% (N = 10) did not have fatigue (mean FACIT-fatigue score, 46.2 ± 2.2). We compared sleep quality, depression, pain, and QOL between in patients with NMSOD with and without fatigue (Fig 1). The mean PSQI was higher in patients with NMOSD and fatigue than in those without fatigue (7.7 ± 3.5 and 4.2 ± 2.2, *p* = 0.009); moreover, poor sleepers (PSQI>5) were more common in patients with fatigue than those without fatigue (73.9% and 11.1%, *p* = 0.004). The mean BDI score in patients with NMSOD and fatigue was 17.9 ± 8.5, which was higher compared to that in patients without fatigue (7.8 ± 3.4, *p* = 0.001), and 8 patients with fatigue (22.9%) showed BDI score ≥ 20, suggesting moderate or severe depression. Pain severity index was higher in patients with fatigue but not significant (3.5 ± 2.3 *vs*. 2.4 ± 2.7, *p* = 0.225). Physical and Mental component scores of SF-36 for QOL were lower in patients with NMSOD and fatigue (*p* = 0.033 and *p* = 0.004) (Fig 1).

**Table 2:** Fatigue, sleep, depression, pain, and health-related quality of life in patients with NMOSD between fatigue groups.

	Total (N = 35)	NMOSD without fatigue (N = 10)	NMOSD with fatigue (N = 25)	*p*
FACIT-fatigue, (SD)	32.9 (13.4)	46.2 (2.2)	27.6 (12.2)	
Sleep quality				
PSQI, (SD)	6.7 (3.5)	4.2 (2.2)	7.7 (3.5)	0.009
Poor sleeper (PSQI > 5), n (%)	18/32 (56.3)	1/9 (11.1)	17/23 (73.9)	0.004
Depression (BDI), (SD)	14.8 (8.7)	7.8 (3.4)	17.9 (8.5)	0.001
Pain (BPI)				
Pain severity index, (SD)	3.2 (2.4)	2.4 (2.7)	3.5 (2.3)	0.225
Health-related quality of life (SF-36)				
Physical component score, (SD)	37.9 (10.7)	43.9 (9.9)	35.4 (10.1)	0.033
Mental component score, (SD)	42.9 (12.2)	51.9 (11.1)	39.1 (10.8)	0.004

NMOSD, Neuromyelitis Optica Spectrum Disorder; FACIT fatigue, Functional Assessment of Chronic Illness Therapy-fatigue; SD, standard deviation; PSQI, Pittsburgh Sleep Quality Index; BDI, Beck Depression Inventory; BPI, Brief Pain Inventory; SF-36, Short-Form 36 Health Survey.

**Fig 2:**
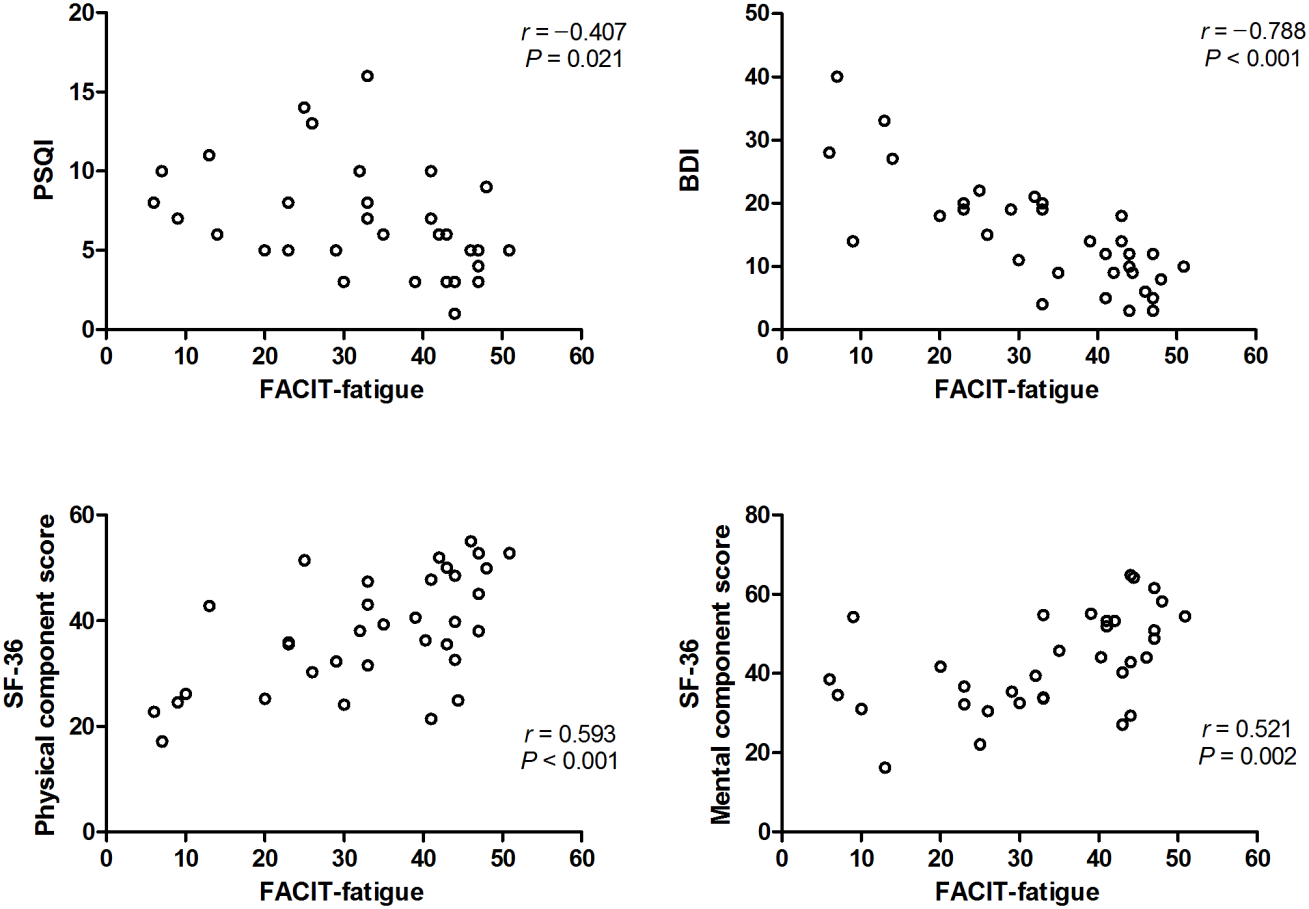
Scatter plots of correlations among PSQI, BDI, the physical and mental component scores of SF-36, and FACIT-fatigue. There were negative correlations among PSQI score, BDI score, and FACIT-fatigue score. Furthermore, the physical and mental component scores of SF-36 were positively correlated with FACIT-fatigue score. PSQI, Pittsburgh Sleep Quality Index; BDI, Beck Depression Inventory; FACIT-fatigue, Functional Assessment of Chronic Illness Therapy-fatigue; SF-36, Short-Form 36 Health Survey.

PSQI score, pain severity index, and BDI score were negatively correlated with FACIT-fatigue score (r = –0.407, *p* = 0.021; r = –0.335, *p* = 0.049; and r = –0.788, *p* < 0.001), whereas the degree of fatigue was not correlated with EDSS score or disease duration (*p* = 0.139 and *p* = 0.667, respectively) (Fig 2). In addition, both the physical and mental component scores of SF-36 were well correlated with FACIT-fatigue score (r = 0.593, *p* < 0.001 and r = 0.521, *p* = 0.002). Moreover, multiple stepwise linear regression analysis showed that EDSS score (B = -1.485, *p* = 0.019), FACIT-fatigue (B = 0.382, *p* = 0.001), and Pain severity index (B = -0.845, *p* = 0.010) were independent predictors of the physical aspects of QOL (R^2^ = 0.651), whereas BDI score was the only predictor of the mental components of QOL (B = -0.845, *p* = <0.001, R^2^ = 0.365) (Table 3).

**Table 3:** Multiple linear regression analysis of factors affecting quality of life in patients with NMOSD.

	Predictors^a^	B	*p*	R^2^
SF-36 Physical component score	EDSS	-0.302	0.019	0.651
	FACIT-fatigue	0.460	0.001	
	Pain severity index	-0.347	0.010	
SF-36 Mental component score	BDI	-0.604	<0.001	0.365

EDSS, Expanded Disability Status Scale; FACIT-fatigue, Functional Assessment of Chronic Illness Therapy-fatigue; SF-36, Short-Form 36 Health Survey; BDI, Beck Depression Inventory.

^a^Age, disease duration, EDSS, FACIT-fatigue, BDI, PSQI, and Pain severity index were included as possible independent variables for multivariate analysis.

## Discussion

This study showed that fatigue was common and an important predictor of QOL in patients with NMOSD. In addition, higher fatigue severity was associated with severe depression, poor sleep quality, and severe pain intensity.

Fatigue is a widespread symptom in various neuroimmunological diseases such as MS, myasthenia gravis, Behcet disease, neurosarcoidosis, and immune vasculitis.[17] Recently, it was reported that chronic fatigue was equally prevalent in patients with NMO (76.9%) and MS (70.9%).[18] Another study showed that the incidence of fatigue was higher in patients with NMO (64%), compared to healthy controls (35%).[19] Our results are consistent with these studies; the frequency of fatigue was 71.4% (N = 25/35).

Generally, 2 types of fatigue exist in patients with neurological disorders. Central fatigue is considered as a characteristic of hypothalamic-pituitary-diencephalic syndrome; metabolic and structural lesions that disrupt the pathways interconnecting the basal ganglia, thalamus, limbic system, and higher cortical centers are implicated in the pathogenesis of fatigue.[20] Previously, it was reported that patients with seropositive NMOSD and fatigue had MS-like brain lesions mainly located in the frontal and parietal periventricular regions.[19] In our study, patients with and without fatigue did not differ with respect to the presence of frontal or parietal lesions; the lesions located at the diencephalon (N = 3), corpus callosum (N = 7), corticospinal tracts (N = 6), and cerebellum (N = 5) were detected only in patients with fatigue, whereas those lesions were not found in patients without fatigue (S1 Table). Another fatigue, peripheral type occurs in myopathy, defects in neuromuscular junction, or peripheral neuropathy, which causes muscle fatigability with or without increased creatine kinase. The binding of AQP4-ab to AQP4 on the sarcolemma of muscle fibers can lead to muscle injury and leakage of creatine kinase by complement-mediated cytotoxicity.[21, 22] However, myopathy or muscle fatigue during the disease course was not observed in our patients. More interestingly, both muscular fatigue and central fatigue exist after spinal cord injury, as in post-polio syndrome.[23] Although the frequency of spinal cord involvement and the length of cord lesions were not different between patients with and without fatigue, the involvement of both the brain and spinal cord was observed only in patients with fatigue (N = 10, 28.6%), but not in those without fatigue (N = 0) (*p* = 0.034). And the patients with ‘myelitis only’ showed more fatigue (lower FACIT-fatigue score) than those with ‘optic neuritis only’, but which was not significant (9 patients with TM-only *vs*. 5 patients with ON-only; 30.3 ± 13.2, 41.4 ± 10.5; *P* = 0.112). Recently, Magnetic Resonance Imaging (MRI) studies in patients with MS showed that grey matter pathology or occult white matter damage was associated with the pathogenesis of fatigue.[24–26] Since the characteristics of brain lesions in NMOSD are quite different with those in MS,[27–29] further MRI studies might help to elucidate the mechanism of fatigue in NMOSD.

Fatigue in NMOSD also could be secondary to sleep disorders, mood disorders or medications. In this study, we found that patients with fatigue had poorer sleep quality and more severe depression than patients without fatigue, and that fatigue score was correlated with sleep quality and depression scores and pain severity index. A previous study showed that patients with fatigue had higher PSQI and BDI scores with more daytime sleepiness and lower blood oxygen level in polysomnography than observed in patients without fatigue.[19] But considering the frequent brain involvement of ependymal regions surrounding the third ventricle and cerebral aqueduct in NMOSD, it is conceivable that patients with NMOSD can have sleep disorders. Hypersomnia and narcolepsy were reported in patients with NMOSD and hypothalamic lesions, one of the brain lesions characteristic of NMOSD.[29–31] And a recent study of Song et al. showed that patients with NMOSD had disrupted sleep architectures (high wake time after sleep onset, low sleep efficiency and decreased slow-wave sleep), and that infratentorial lesions were associated with frequent periodic leg movements during sleep.[32] In our study, only 3 patients showed hypothalamic and diencephalic lesions; however, all of them showed very poor sleep quality as well as severe fatigue (2 female and 1 male; FACIT-fatigue score, 19.3 ± 18.8; PSQI, 10.3 ± 0.6). Therefore, sleep abnormalities could be one of clinical features of NMOSD and important for causing fatigue in patients with NMOSD; the coexistence of sleep disorders should be considered firstly for treating fatigue in patients with NMOSD.[33] Further studies of brain lesions-related effects on sleep abnormalities in NMOSD are needed.

Regarding depression, it was reported that patients with depression showed a significantly higher cortisol awakening response, indicative of hypothalamic-pituitary-adrenal (HPA) axis hyperactivity than healthy subjects.[34] The relationship between fatigue, sleep, and depression may suggest of the common mechanism, the dysregulation of HPA axis, which is sensitized by stress.[18–20] In patients with NMOSD, pain scores were correlated with the depression scores; however, they were confounded by fatigue scores.[35] The degree of pain, assessed by a subscale of MS quality of life (MSQOL) was also correlated with the fatigue impact scale in patients with NMO.[35, 36] A previous study suggested that pain may be influenced by spinal cord pathology in patients who were seropositive for anti-AQP4.[37] However, it remains to be further investigated whether HPA axis dysregulation could contribute to the development of pain in NMOSD, although HPA axis dysfunction has been observed in MS as well as several chronic pain states.[38] On the other hand, the degree of fatigue was not correlated with disease disability or disease duration; patients had fatigue even in an early stage (S1 Fig), and steroid or immunosuppressant use, and age were not associated with fatigue in our patients. This was not congruous with previous results showing that longer disease duration would contribute to the advent of chronic depression and fatigue in NMO, although oral steroid dose and present age were not associated with the scores of both depression and fatigue.[18]

More importantly, we found that fatigue score was correlated with both the physical and mental scores of SF-36, and that fatigue, pain, and EDSS scores were independent predictors of the physical aspects of QOL, whereas depression was an independent predictor of the mental aspects of QOL. Previously, it was reported that very strong correlation was observed among global fatigue, global depression, and decrease in HRQOL scores in patients with NMO,[8] and recent reports also suggested that pain had a grave impact on the QOL in patients with NMOSD.[37, 39, 40] Furthermore, high EDSS scores were suggested as the main predictive factor of impairment of QOL, fatigue, and depression.[8] Therefore, fatigue, depression, and pain might be important aspects of treatment for the improvement of QOL in patients with NMOSD.

This study has several limitations. First, we included a single ethnic population at a single hospital, which can result in unintentional bias. In addition, only patients who were seropositive were enrolled because we did not perform the test of myelin oligodendrocyte glycoprotein (MOG) autoimmunity, a distinct spectrum differentiated from seronegative NMOSD, which can limit the generalization of our data to seronegative NMOSD.[41, 42] Finally, other factors including socioeconomic variables that can affect fatigue or QOL,[43] were not investigated in this study, although we completed the comprehensive investigations of possible contributing factors of QOL (fatigue, sleep quality, depression, and pain), in remission.

In conclusion, fatigue is a common symptom and an important predictor of QOL in patients with NMOSD. The positive correlation of fatigue with depression, sleep disturbance, or pain may suggest that the mechanisms of these factors are interactively associated in NMOSD. The management of fatigue with the efforts to treat depression, poor sleep quality, and pain could help to improve QOL in patients with NMSOD.

## Supporting information

S1 Table Spine and brain Magnetic Resonance Imaging characteristics of patients with or without fatigue. (DOCX)

S1 Fig Correlations among EDSS, disease duration, and FACIT-fatigue. The degree of fatigue was not correlated with disease disability or disease duration. The dotted line represents the FACIT-fatigue score of 43. EDSS, Expanded Disability Status Scale; FACIT-fatigue, Functional Assessment of Chronic Illness Therapy-fatigue.
(TIF)
