# Supplementary material for: Fatigue in patients with neuromyelitis optica spectrum disorder and its impact on quality of life
Source: PLoS One. 2017 May 23;12(5):e0177230. doi: 10.1371/journal.pone.0177230 (PMC5441592; doi:10.1371/journal.pone.0177230)
Supplement: S1 Table — (DOCX) [file pone.0177230.s001.docx]

|  | Total  (N=35) | NMOSD  without fatigue  (N=10) | NMOSD  with fatigue  (N=25) | *p* |
| --- | --- | --- | --- | --- |
| Longitudinal length of spinal cord lesion, (IQR) | 5.3 (3.0-10.0) | 3.0 (2.0-7.5) | 5.5 (4.0-12.5) | 0.224 |
| White matter lesion, n (%) |  |  |  |  |
| Frontal | 10 (28.6) | 2 (20.0) | 8 (32.0) | 0.686 |
| Parietal | 9 (25.7) | 2 (20.0) | 7 (28.0) | 1.000 |
| Non-specific lesions | 14 (40.0) | 4 (40.0) | 10 (40.0) | 1.000 |
| Diencephalic lesion, n (%) | 3 (8.6) | 0 (0) | 3 (12.0) | 0.542 |
| Periependymal lesion around lateral ventricle, n (%) | 12 (34.3) | 4 (40.0) | 8 (32.0) | 0.706 |
| Callosal lesion, n (%) | 7 (20.0) | 0 (0) | 7 (28.0) | 0.084 |
| Corticospinal tract lesion, n (%) | 6 (17.1) | 0 (0) | 6 (24.0) | 0.152 |
| Dorsal brainstem lesion, n (%) | 5 (14.3) | 1 (10.0) | 4 (16.0) | 1.000 |
| Cerebellar lesion, n (%) | 5 (14.3) | 0 (0) | 5 (20.0) | 0.292 |

NMOSD, Neuromyelitis Optica Spectrum Disorder; IQR, Inter-Quartile Range.
